# 3D printed laboratory equipment to measure bulk materials in extreme conditions

**DOI:** 10.1038/s41598-022-22114-2

**Published:** 2022-10-15

**Authors:** Jan Divis, Jakub Hlosta, David Zurovec, Jiri Rozbroj, Weronika Kruszelnicka, Jan Necas, Jiri Zegzulka

**Affiliations:** 1 ENET Centre, Bulk Solids Centre, VSB-TU Ostrava, 17. listopadu 15/2172, 708 33 Ostrava-Poruba, Czech Republic; 2 Department of Mining Engineering and Safety, Faculty of Mining and Geology, VSB-TU Ostrava, 17. listopadu 15/2172, 708 33 Ostrava-Poruba, Czech Republic; 3 Department of Machines and Technical Systems, Faculty of Mechanical Engineering, Bydgoszcz University of Science and Technology, Al. Prof. S. Kaliskiego 7, 85-796 Bydgoszcz, Poland

**Keywords:** Mechanical engineering, Mechanical properties, Characterization and analytical techniques

## Abstract

Due to relatively new solutions in the field of 3D printing, there are few studies on the possibility of using printed elements in measuring devices. The aim of this study was to investigate the possibility of using instruments made by material extrusion 3D printing method for measurement of selected mechanical-physical properties of bulk materials. Study explores the feasibility of measuring bulk material mechanical-physical properties when there are obstacles for printing original or modified measuring instruments in common practice. To achieve the goals a series of experiments such as Schulze’s ring shear tests, Freeman’s FT4 shear tests, compressibility tests, and Flow Rate and Stability tests were performed with use of original aluminium or steel made instruments and 3D printed instruments from polylactic acid and acrylic styrene acrylonitrile materials, using lunar regolith simulants LHS-1 and LMS-1 produced by CLASS Exolith Lab as a sample material. The results obtained from tests with original and printed instruments were then compared. The compared values of tests showed applicability of the 3D printed measuring instruments in a 5% range of measurement deviation. The biggest advantages of the 3D printed measuring instruments were the lower weight, the ability to print on the spot, to replace a damaged part with a new 3D printed part on-demand if extremely fast results are needed or due to the logistical unavailability, customization of the standardized tests for better understanding the behaviour of the particulate materials, and cheaper manufacturing costs.

## Introduction

Scientists and engineers made significant development in the exploration missions of planets and celestial bodies in last few decades and gained knowledge about their resources and their properties. However, besides reaching the planets, to land safely in the universe still proves to be a difficult task. To change this, geology resources, atmosphere and radiation data are gathered by landers and rovers, which are required to verify measurements by probes from orbit. Landers and rovers provided with excavator booms extract rocks and dust for material properties analysis^1^. The aim is to gather data and prepare strategies to build landing sites and radiation shielding habitats, and to develop suitable constructions, such as infrastructure, factories, and laboratories, prior to the arrival of astronauts.

To extend and facilitate such exploration missions, two in situ concepts are needed^2,3^. Firstly, it is in situ fabrication and repair (ISFR) equipment and infrastructures. Secondly, it is in situ resource utilization (ISRU). As a result, resources for in situ lunar fabrication have been studied intensely in the last decade and several technologies have been proposed^4–7^. To simulate materials on other planets, ceramic-based products are used, such as lunar regolith^1^, which is very fine sand^8^. In terrestrial environment lunar regolith simulants with similar mechanical-physical properties^9^ were developed, such as LHT-1 M^3^, NU-LHT^7^ or JSC-1A^10^. However, due to different physical environment, material properties and behaviour on other celestial bodies differ from Earth. Behaviour of real regoliths differs based on the linearized angle of internal friction (*LAIF*, *ϕ*), effective angle of internal friction (*EAIF*, *δ*), flow function (*ff*_*c*_), cohesion *c*, and compressibility, depending on environment which regoliths are measured in, place of regolith excavation, environment of regolith origin and environment of regolith transformation. The composition of regoliths varies from place to place because of the variability in asteroid collisions and the weathering by wind or water. Therefore, there will be a crucial need to be able to measure mechanical-physical properties of in situ regoliths and bulk material resources during the exploration missions^11^.

Due to the fact that transportation of any equipment from Earth is very costly, currently it may take years to get spare parts to orbit. This problem has been partially overcome by fused deposit modelling (FDM, registered trademark by Stratatys) technology modified for microgravity^12^. FDM is a type of additive manufacturing (AM), where a 3D geometry is built by superimposed layers of extruded thermoplastic filament^13^. FDM technology modified by Made in Space projects^14,15^ explore the possibility to create tools^16^ that astronauts currently need for repairs or work. FDM allows for the use of a broad range of thermoplastics^13^ which are light but durable and can withstand a certain extent of mechanical load when designed properly. FDM printing is also highly precise and most of its advantages are due to the enclosed printing chamber which allows the internal temperature to be maintained (nozzle-air-heated bed). It leads to better mechanical properties, where the adhesion between layers is strengthened and warping and curling of the printed parts are prevented^16^. However, the technology is very costly and is not widely available for research. Extending the ability and options to print parts on demand in orbit or during exploration missions will reduce the time it takes to get parts to orbit, reduce the mission costs, reduce the need of having every tool and part on board, while increasing the reliability and safety of space missions.

Despite the developments in the field of 3D printing, there is a lack in studies on the use of printed elements in measuring devices and/or devices intended to measure bulk materials. Traciak et al.^17^ developed a 3D printed device to measure the surface tension of nanofluids and showed that the result of measurement is comparable with commercial devices. Bernard and Mendez^18^ presented a low-cost Polarimeter to be used by students during classes. The study^19^ described the dynamic behaviour of 3D-printed strain sensors embedded in structures and supported the statement that 3D printed sensors could be used for dynamic measurement. The study^20^ reported the design of a 3D printed compact interferometric system for cell phones to measure small angles. All these studies show a high potential of 3D printing devices and the lack of specific guidance for the manufacturing of measuring equipment.

In order to fill the gap in this area, the aim of this study was to investigate the possibility of using measuring instruments made by material extrusion 3D printing method for the measurement of selected mechanical-physical properties of bulk materials. Due to the unaffordability of FDM 3D printing technology and related problems such as testing the effects of high radiation environments on printed measurement tools, fused filament fabrication (FFF) 3D printing technology was used in this study. This article thus presents a feasibility study of measuring mechanical-physical properties of bulk materials using 3D printed instruments, should the reasons for doing so arise. Lu et al.^21^, Li et al.^22^, and Pelech et al.^23^ showed why is to measure important mechanical-physical properties of lunar regolith simulants. Further, the use of 3D printed instruments to measure standardized mechanical-physical properties of particulate materials is not researched. These reasons for printing original or modified measuring instruments are also encountered on Earth, such as the need for a lower weight of the measuring instruments, the ability to pre-print a set of laboratory measuring instruments or to print the set on the spot, to replace a damaged part with a new 3D printed part on-demand, the logistical unavailability, customization of the standardized tests for better understanding of the behaviour of the particulate materials, and cheaper manufacturing cost. The 3D printed instruments are preferred when versatility of the instruments, light weight, and/or quick result in the extreme locations are needed. If the conditions are laboratory and the measured particulate material is without unusual properties which requires customized measuring tools, standardized tests should be made.

Supposing the measuring instruments will be used for exploration mission, regolith simulant samples were also tested. The measurements of the mechanical-physical properties such as *EAIF* (*δ*), *LAIF* (*ϕ*), *ff*_*c*_, cohesion *c*, compressibility, basic flowability energy *BFE*, stability index *SI*, and flow rate index *FRI* for lunar regolith simulants: lunar mare simulant (LMS-1) and lunar highlands simulant (LHS-1) from the CLASS Exolith Lab in Orlando, USA, are presented. *EAIF* (*δ*), *LAIF* (*ϕ*), *ff*_*c*_, *c*, and compressibility are fundamental characteristics of bulk material flow, which is used to design storage, handling, and process equipment. Firstly, two lunar regolith powders were characterized by particle size distributions and their morphology. Secondly, comparison of results was carried out between standard measuring instruments and 3D printed instruments from polylactic acid and acrylic styrene acrylonitrile materials. Values of *EAIF* (*δ*), *LAIF* (*ϕ*), *ff*_*c*_, *c*, compressibility, *SI*, *FRI*, and *BFE* were compared. Results presented in this article showed repeatability and similar precision for the test methods of Schulze’s ring shear test, Freeman’s FT4 shear test, Freeman’s FT4 compressibility standard test, and Freeman’s FT4 flow rate and stability standard test. This showed applicability of 3D printed instruments for the test methods in hardly reachable, or extra-terrestrial environment.

## Materials and methods

The materials and methods concern two areas. The first area is the materials and methods related to the printed measuring instruments made via Fused Filament Fabrication. The second area of interest is the bulk material (regolith) used to test the produced measuring instruments. The subsection of Bulk Material Tests describes all the tests related to examining the performance of the 3D printed measuring instruments, of the 3D printed measuring instruments in combination with original stainless steel components, and of original stainless steel instruments.

### Fused filament fabrication printed equipment

**Figure 1:**
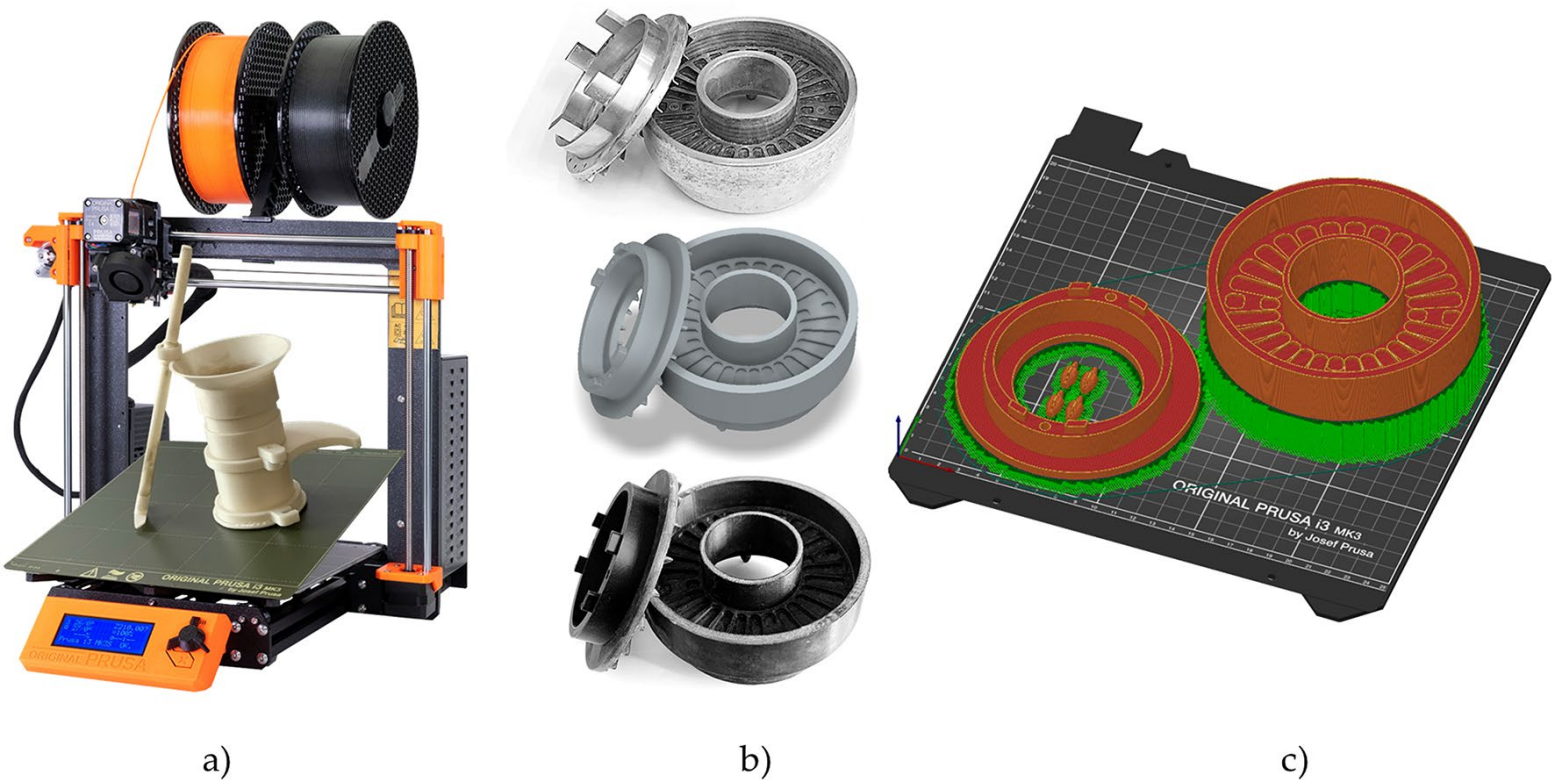
(**a**) Prusa i3 MKS3 printer with FT4’s 3D printed blade and vessel; (**b**) Schulze’s S size shear cell with lid (from top to bottom–aluminium, 3D CAD by Autodesk Inventor 2021, 3D printed); (**c**) Schulze’s shear cell sliced in PrusaSlicer version 2.3.0. This figure was created in Gimp 2.10.32.

The measuring instruments were printed by fused filament fabrication (FFF) 3D printing technology on a Prusa i3 MKS3 printer (Praha, Czech Republic), which is shown in Figure 1a. PLA and ASA filaments manufactured by Prusament were used. ASA filament is the successor of ABS filament with superior properties, such as UV stability, high impact resistance, wear resistance, and easier printability for FFF printing method^24^.

The Prusa i3 MKS3 printer uses G-code numerically controlled machines, which allows users to provide instructions telling the motors where to move, how fast to move, what path to follow and how fast to feed the filament. Before creating the G-code, a 3D CAD model of the original is created that could be 3D printed as shown in Figure 1b. The G-codes for the prints were sliced in PrusaSlicer version 2.3.0 with the layer height of 0.20 mm as shown on Figure 1c. The infill for all the parts had different infills that are shown in this section. The infill pattern was chosen as gyroid except for 100% infill, which is forced to be rectilinear. The PLA instruments were printed at an extrusion temperature of 210 °C and bed temperature of 60 °C. The ASA instruments were printed at an extrusion temperature of 260 °C bed temperature of and 110 °C.

For the Schulze’s RST-01.pc (RST) tests, we used a set of shear cell and lid from different materials (original aluminium, PLA printed, ASA printed). RST tests are described in the following subsection Bulk Material Tests.

For the Freeman’s Flow Tester 4 (FT4) tests, we used a 85 ml measuring sample vessel as an assembly of parts that will contain the sample powder during measurements. The 3D printed sample vessels and instruments were printed from PLA and ASA filaments. These printed instruments required design modifications to withstand mechanical loads. The printed vessels were either all printed or were partly 3D printed and also comprised of original components, such as compression piston and blade manufactured from stainless steel. Overall, we used a set of specimens (original stainless steel, PLA printed, ASA printed) and their combinations (original vessel with a PLA blade, original vessel with an ASA blade, PLA vessel with an original blade, and ASA vessel with an original blade). FT4 tests are described in the following subsection Bulk Material Tests.

**Figure 2:**
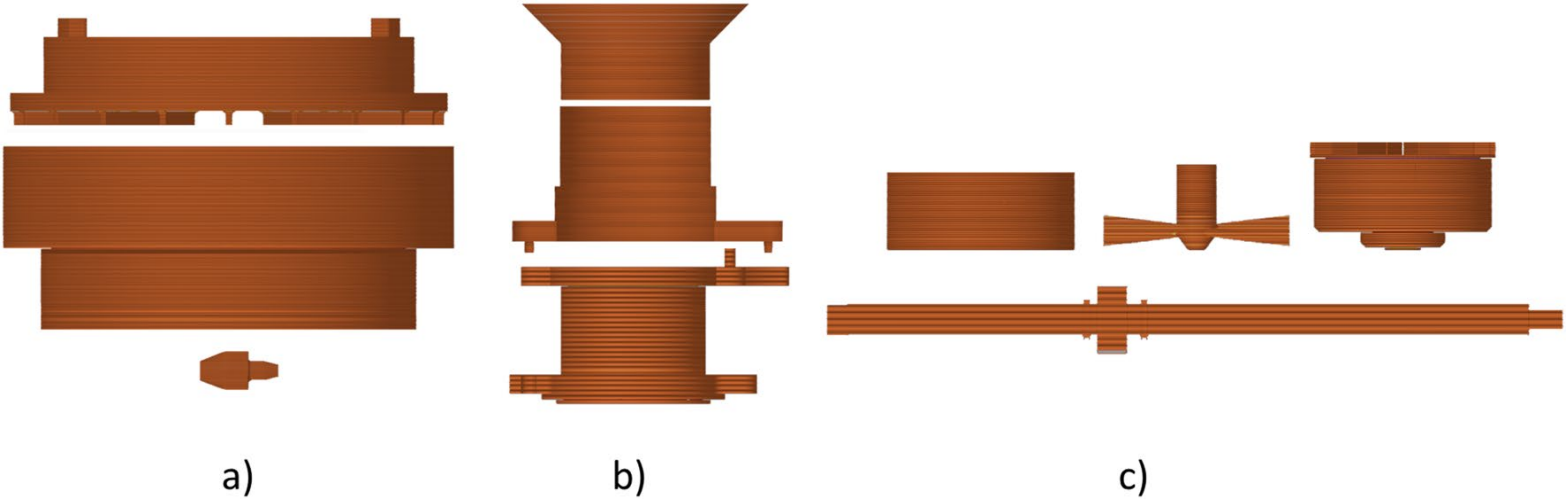
(**a**) The layering of the Schulze’s small ring cell (S size), lid, and driving pins; (**b**) the layering of the FT4’s bottom part, upper part, and funnel; (**c**) the layering of the FT4’s shaft with the nut, shear head, blade, and vented piston. Layering was generated by PrusaSlicer version 2.3.0. This figure was created in Gimp 2.10.32.

Fused filament fabrication (FFF) 3D printing technology creates parts layer by layer. A consequence of layering is the presence of pores and heterogeneities that cause anisotropic behaviour and preferential crack orientation^25^. The resistance of parts to mechanical damage is dependent on the orientation of the deposited layers^26–28^. Thus, the orientation of the parts on the 3D printer bed is an important consideration when manufacturing components^29–31^. The orientation of the measuring instruments was chosen accordingly as shown on Figure 2., and the design modifications have been made to the measuring instruments to prevent damage due to mechanical loading. The Figure 2 is illustrative to show layering of the parts from the bottom up and the picture does not show support material, support material interface, skirt, bridge infill, and overhang perimeter. The layers are stacked from the bottom up. The Figure 2a shows Schulze’s small ring cell, lid, and driving pins. The Figure 2b shows layering of the FT4’s bottom part, upper part, and funnel. The Figure 2c shows layering of the FT4’s shaft with the nut, shear head, blade, and vented piston.

The Schulze’s small ring cell (S size)^32^ was printed with bottom plate as one piece. Three pins with interference fit were printed separately to rotate the cell by driving axle. The Schulze’s lid matching small ring cell was printed as a single piece. The shear bars^32^ were thickened from 1 to 2 mm and no screws were used. The design of the original, the 3D modelled, and the PLA fabricated shearing cell with lid are shown in Fig. 1b. The weighed parts, the weighed printed parts with supports and their infill percentage used in printing are shown in Table 1. The original aluminium shear cell weighed 728.4 g and the original aluminium lid with stainless steel shearing bars weighed 235.4 g. The PLA printed parts weighed 2.6 times less, and the ASA printed cell weighed almost 3 times less than the original parts. Infill used for all Schulze’s printed parts was 100%. The differences in weight are due to different material densities. Aluminium has a density of 2.7 g.cm^−3^, PLA filament has a density of 1.24 g.cm^-3^, and ASA filament has a density of 1.07 g.cm^−3^.

**Table 1:** Original and 3D printed measuring instruments weight, 3D printed parts with supports weight and printed parts infill in percentage as used in PrusaSlicer.

Measuring tool	Original (g)	PLA printed (g)	PLA filament used (g)	ASA printed (g)	ASA filament used (g)	Printed part infill (%)
Schulze’s shear cell	728.4	283.1	347.8	244.4	300.1	100
Schulze’s lid	235.4	89.5	107.7	77.5	92.9	100
FT4’s compressibility vessel (two 85 ml vessels, removable bottom, holder of the vessel, rotational holder for upper part)	291.8	92.8	134.9	80.2	123.3	10
FT4’s internal friction vessel (two 85 ml vessels, removable bottom for internal friction, holder of the vessel, rotational holder for upper part)	289.9	109.8	159.0	94.9	137.2	10
FT4’s flow stability vessel (one 85 ml vessel, one 165 ml vessel, removable bottom, holder of the vessel, rotational holder for upper part)	345.7	110.2	190.8	95.0	164.7	10
FT4’s funnel	30.7	25.8	31.6	22.2	27.3	10
FT4’s blade	111.8	13.6	17.9	10.9	15.5	7 (shaft)/15 (head)
FT4’s vented piston	184.0	30.6	36.5	26.3	31.5	7 (shaft)/15 (head)
FT4’s shear head	240.9	25.7	35.0	21.5	30.2	7 (shaft)/15 (head)

**Figure 3:**
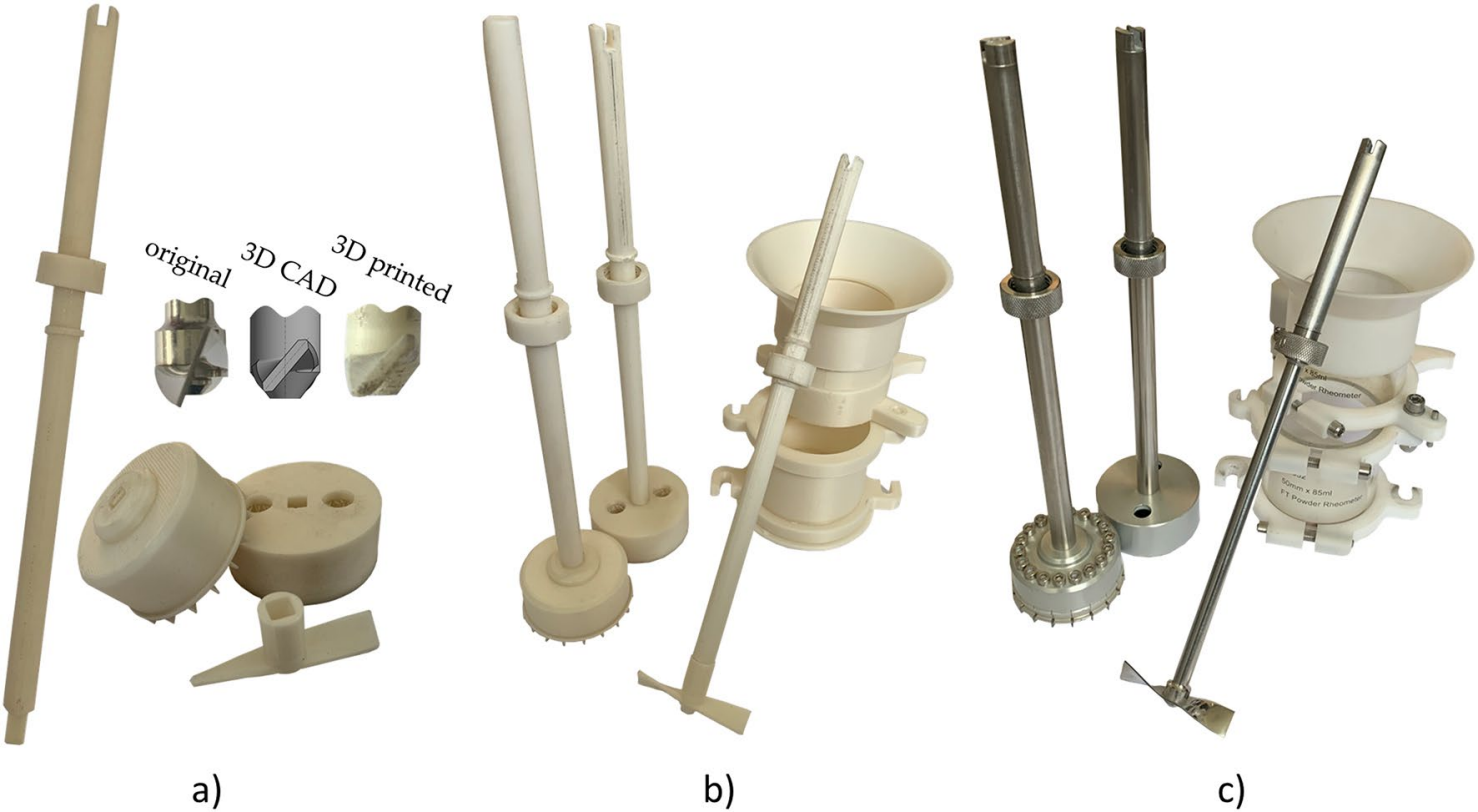
Design of FT4 instruments (**a**) FT4’s printed shaft, blade, vented piston, and shear head; (**b**) PLA printed FT4’s measuring set; (**c**) original FT4’s measuring set. This figure was created in Gimp 2.10.32.

The 3D printed FT4 measuring set and the original measuring set are shown in Figure 3b and c. FT4’s sample vessel is originally made from five pieces, which were reduced to two parts. The 3D printed bottom part has replaced 85 ml vessel with an inner diameter of 48 mm, its removable bottom, the holder that keeps it in place during measurement, and rotational holder for top part. The 3D printed upper part replaced for the upper 85 ml vessel with an inner diameter of 48 mm and it is pivotally seated on the bottom part. The original vessel assembly of two 85ml vessels with a removable bottom, a holder that keeps the whole vessel in place during measurement, and rotational holder for the upper part weighed 291.8 g. The original vessel assembly for angle of internal friction measurement differed by having a removable bottom for internal friction and weighed 289.9 g. Vessel assemblies printed from PLA for compressibility and internal friction measurements weighed approximately 3 times less than the original assemblies. The ASA printed vessel assemblies weighed more than 3.3 times less than original assemblies.

The 3D printed funnel had a reduced height, which does not affect the measurement. The original funnel is made of plastic and weighed 30.7 g. The PLA printed funnel weighed 25.8 g, and the ASA printed funnel weighed 22.2 g.

The FT4 measuring instruments such as the shear head, blade, and vented piston, were 3D printed in two parts. The first part was the shaft with the nut, which was the same for all three measuring instruments. The shaft was connected to the second part by end of a square cross-section shaft (Figure 3a). This shaped connection transmits torque without the two parts of the measuring tool rotating against each other. The manufacturing precision of the FFF printing method created a slight overlap that allowed the two parts to be joined by hand but did not require protection against ejection. The shaft infill was chosen as 7% to overcome the warping during printing with ASA filament. The infill setup improves geometric dimensions and tolerances, such as radial runout and total run out of the shaft. The second parts of the blade, the vented piston, and the shear head were printed with 15% infill setup. Design modifications to the instruments were made to prevent damage due to mechanical loading, the shearing bars of the shear head were thickened from 0.1 to 0.8 mm, and the blade was thickened from 0.7 to 1.8 mm. The 3D printed shear head was printed as one piece, so the screws were not used in the design. The blade was further modified to ensure similar forces and torques during conditioning of the measured samples. The curvature of the blade had less bending, resulting in a smaller angle at each end of the blade (Figure 3a). The original blade had the end of the blade curved at an angle of 70 degrees, while the end of the 3D printed blade is only curved at an angle of 40 degrees.

The price of 3D printing has its benefits. The material for 3D printing (PLA and ASA) is approximately 3 times more expensive compared to aluminium or stainless steel per kilogram. However, 3D printed instruments are 2.5 to 10 times lighter than the originals. After accounting for productional waste, the difference in material weight is even greater. The costs diversify depending on the cost of machining, complexity of machining, need to change machining tools. In contrast, 3D printing is more versatile, simpler, and with lower weight of the final product. The productional costs of the original instruments are at least 30 times higher than for 3D printed instruments.

### Bulk material

Regolith is a terrestrial term which is also used to refer to materials on other celestial bodies. Nowadays, it is used as a common expression for a layer of fragmental rock material. The formation and evolution of regolith is a complex process. In the formation of lunar regolith, two basic mechanisms have been determined. Firstly, destructive, which is the excavation of existing regolith by impact crater, and secondly, constructive, which is the addition of new layers. These processes cause very wide structural and stratigraphical differences in regolith, even between locations only few meters apart^11^.

**Figure 4:**
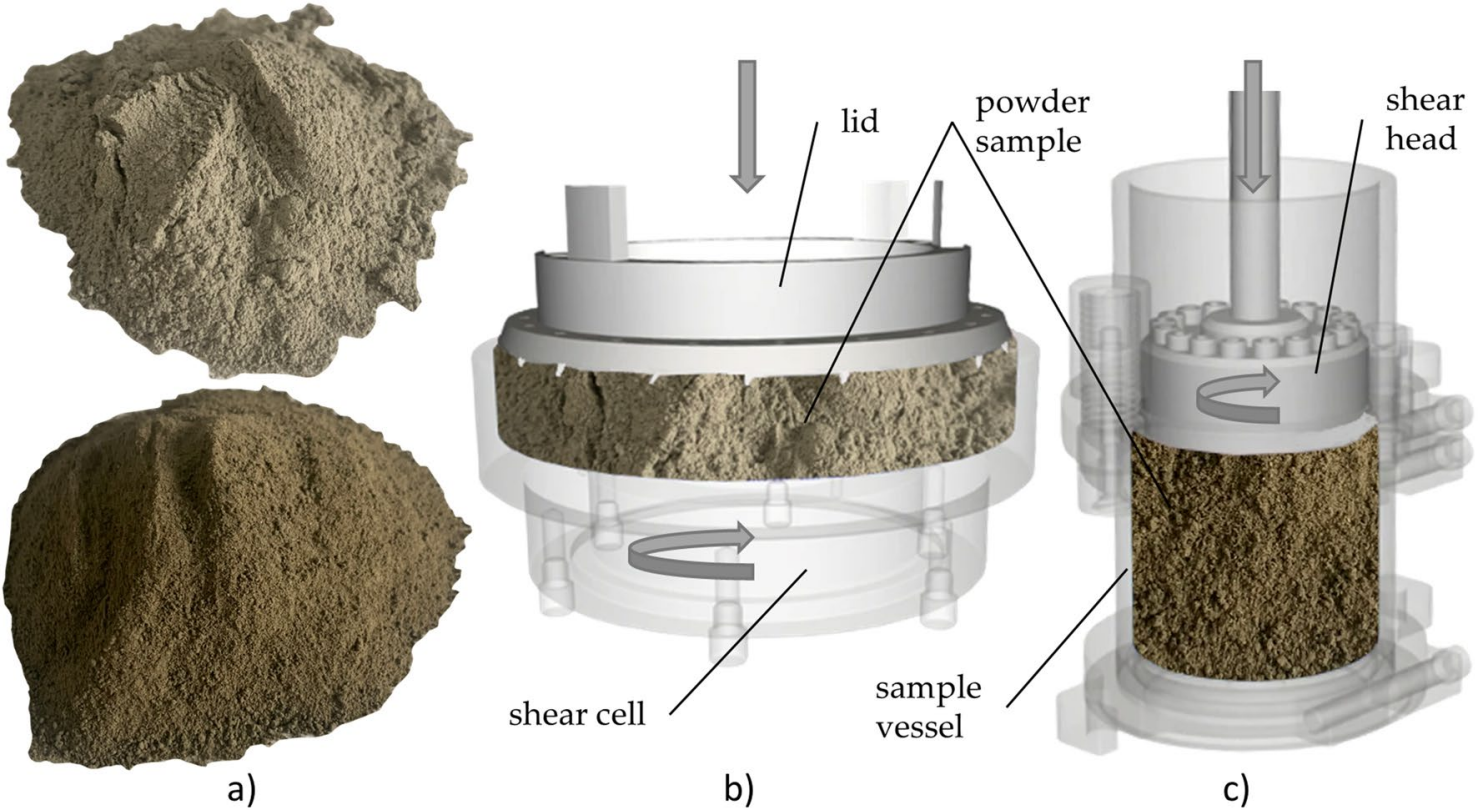
(**a**) Lunar regolith simulant LHS-1 (above) and LMS-1 (bottom); (**b**) Schulze’s shear test setup; (**c**) FT4’s shear test setup. This figure was created in Gimp 2.10.32.

Lunar regolith simulant powders are terrestrial based on samples analysed by experiments carried out directly on the Moon, or remotely monitored from Earth^11^. As mentioned above, materials used in this study are two lunar regolith simulants. These two powders were made by the CLASS Exolith Lab. The simulants, shown on Fig. 4a, are made from natural terrestrial materials in a terrestrial environment, and thus not all properties of lunar mare simulant (LMS-1) and lunar highlands simulant (LHS-1) may be copied. The producer guarantees properties such as mineralogy, bulk chemistry, and particle size distribution. However, particle shape, reactivity, oxidation, and weathering are poorly simulated properties in the simulants.

Although the particle size is guaranteed by the manufacturer to be between 0 and 1 mm, a granulometric analysis was carried out. Cilas 1190 laser analyzer (Cilas, Orleans, France) was used to measure the particle size distribution by the Fraunhofer diffraction method^31^. Water was used as the measurement medium because neither LHS-1 nor LMS-1 dissolved in water. Sonication was used during the measurements to ensure complete dispersion of the sample. The sample dispersed in medium was measured using a coherent light with a wavelength of 830 nm from a low-power laser diode. The resulting values were evaluated directly in the Cilas device^33,34^. The interpretation was based on Fraunhofer's theory^35^. Each sample was measured three times, so the resulting parameters are the average values of d_mean_, d_10_, d_50_ and d_90_.

Particle shape is a property of a bulk material that affects its behaviour during extraction, processing, and storage. As mentioned above, lunar regolith simulant manufacturer does not attempt to simulate exact particle shapes. The lunar regolith is formed by various processes that are the constant impacts of small and large asteroids and that are unique to the airless conditions of the Moon^11^. Therefore, the assessment of particle shapes presented in this paper is only illustrative based on scanning electron microscope (SEM) photographs.

### Bulk material tests

The following tests were used to test the performance of the printed measuring instruments. To verify the measurement stabilty of the printed instruments, we also used 3D printed instruments in combination with original stainless steel instruments, and original steel instruments.

### Shear test

Shear properties show how easily particulate material flows. For a particle material flow to occur, the yield point must be overcome. The yield point is greatly influenced by mechanical-physical properties of the particles such as surface properties, shape, and size. Other variables, like moisture content, Van der Waals forces, or level of flow additive also affect the flow of bulk material. The shear properties of bulk materials are used in the design of handling, storage, and process equipment.

The flow properties of bulk materials are used in various applications^36^, usually quantified as linearized angle of internal friction (*LAIF*, *ϕ*), effective angle of internal friction (*EAIF*, *δ*), and flow function *ff*_*c*_ to describe the bulk material behaviour^37^. The values of *EAIF* (*δ*), *LAIF* (*ϕ*), *ff*_*c*_, and cohesion *c* were measured on Schulze Ring Shear Tester RST-01.pc (Wolfenbuttel, Germany, Fig. 4b and Freeman’s FT4 Powder Tester (Freeman Technology, Tewkesbury, Gloucestershire, UK, Fig. 4c^38^. The main monitored parameters are *EAIF* (*δ*), *LAIF* (*ϕ*), cohesion *c*, and flow function *ff*_*c*_^39,40^.

The measurements of *AIF* (*δ*, *ϕ*)*, ff*_*c*_, and *c* had pre-shear normal stress of 10 000 Pa, shear points with normal stresses values of 250 Pa, 500 Pa, 1 000 Pa, 2 500 Pa, 5 000 Pa and 7 500 Pa. The resulting values of *AIF* (*δ*, *ϕ*), *ff*_*c*_, and cohesion *c* were the average of ten measurements. Twelve combinations of measurements were performed for two measuring devices, two lunar regolith simulants (LHS-1 and LMS-1), and three measuring set materials (steel and additive plastic materials) giving a total of 120 measurements. The methods of measuring *AIF* (*δ*, *ϕ*), *ff*_*c*_, and *c* were rotational. However, there were different measurement procedures and characteristics in the shear cell. A recent comparison showed that in most cases, lower values of *EAIF* (*δ*), *LAIF* (*ϕ*) and *ff*_*c*_ are obtained using FT4^36^. Due to the incomparable geometry, area ratios, and cell sizes, the total sample volume differs. In general, this behaviour is derived from the property of bulk materials that the bulk material flows less easily through smaller cross-sections.

Schulze’s ring shear cell and lid, and FT4’s sample vessel, funnel, blade, vented piston and shear head were 3D printed from PLA and ASA filaments. The printed instrument designs and their modifications are described in the section Fused Filament Fabrication printed equipment. All 3D printed parts were appropriately calibrated prior to measurement because they exerted less pressure due to their lower weight.

### Compressibility

Compressibility is a property of bulk materials that shows the change in the bulk density as a function of consolidation pressure. The compressibility measurement is neither a shear property nor a flow property of the bulk material but it is dependent on similar quantities. This property is affected by particle size distribution, cohesion, particle surface texture, particle shape, and particle stiffness. Compressibility is an important property for the design of process equipment such as silos, conveyors, mixers, compacting equipment, and tablet presses^41^.

Compressibility was measured with Freeman Technology's FT4 powder rheometer with standard compressibility test^42^. The standard test obtained data by expressing the percentage compressibility for a normal load from 0.5 to 15 kPa applied onto the sample. Samples of lunar regolith simulants were measured in the 85 ml sample vessel with a diameter of 50 mm. Vented compression piston with diameter of 47.5 mm and blade with 48 mm were used.

These design modifications were described in the section Fused Filament Fabrication printed equipment. For each set of instruments (original stainless steel, PLA printed, ASA printed), 10 measurements were performed.

### Stability index, flow rate index and basic flow energy

Stability Index (*SI*), Flow Rate Index (*FRI*) and Basic Flowability Energy (*BFE*) were analysed by using Freeman’s FT4 powder rheometer in a manner that previously described (Freeman Technology, Tewkesbury, Gloucestershire, UK)^42^. The SI, FRI and BFE measurements are performed using the standardized test preset in the FT4 powder rheometer. The *SI* and the *FRI* measurements were carried out in a 65 ml vessel. Five measurements were taken for each set of instrument materials (original stainless steel, PLA printed, ASA printed) and their combinations (original vessel with PLA blade, original vessel with ASA blade, PLA vessel with original blade, and ASA vessel with original blade).

Stability Index (*SI*) of a powder^42^ shows the conditioned flow properties under the action of forces during flow, which may change due to the tendency of powder to agglomeration, caking, and attrition. The *SI* program measures the particulate material by conditioning sequence followed by a test cycle. The test cycles are repeated seven times. The seven measurement points form a straight line, and the more stable the powder, the straighter the line. The *SI* is defined as the ratio of the energy consumed during test 7 to the energy consumed during test 1^43^. The more the *SI* approaches 1, the more stable the measured powder is. If the *SI* > 1, the measured powder is affected by moisture absorption, segregation, agglomeration, de-aeration, and electrostatic charge. If the *SI* < 1, then the measured powder is affected by over-blending, de-agglomeration, attrition, and additive coating of the blade and of the vessel^42^.

The variable FRI^42^ is measured as a decreasing flow in measuring points 8 to 11, where the Flow Rate of the blade decreases from 100 mm.s^-1^ to 10 mm.s^-1^. The FRI of the blade indicates the sensitivity of the measured powder, and it is expressed as Flow Rate Index (*FRI*). Non-cohesive powders show fewer sensitive changes for the *FRI*, which is defined as ratio of the energy test 11 to the energy test 8^42^. The *FRI* < 1 has powders with extremely good flow. The *FRI* = 1 has powders with a surface coating or large particle size distribution, which makes them insensitive to changed flow rate. Most measured powders have Flow Rate sensitivity 3 > *FRI* > 1.5. If the *FRI* > 3, then the powder is overly sensitive to changed flow rate^43^.

The Basic Flowability Energy (*BFE*)^42^ is a property defined by the energy consumed for point 7 during the standardized variable flow test, which corresponds to the flow energy^43^. The energy consumed by the specific flow is generated in the exact volume of the vessel as the blade moves downward.

## Results

### Particle characterization

**Figure 5:**
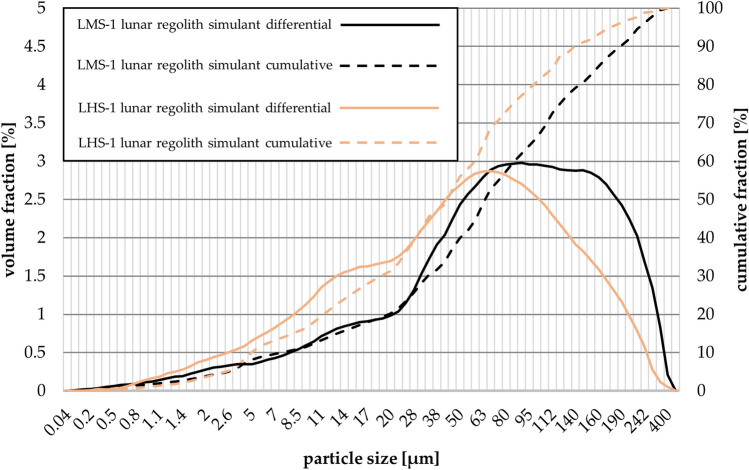
The particle size distribution (differential and cumulative) for LMS-1 and LHS-1 regolith simulants.

The particle size distributions of the regolith simulants LHS-1 and LMS-1 are shown in Figure 5 and the values of d_mean_, d_10_, d_50_, and d_90_ are given in Table 2.

**Table 2:** Particle size distribution characterization values.

lunar regolith simulant	d_mean_ (µm)	d_10_ (µm)	d_50_ (µm)	d_90_ (µm)
LMS-1	86.85	7.62	67.58	193.44
LHS-1	58.71	4.95	41.79	139.32

**Figure 6:**
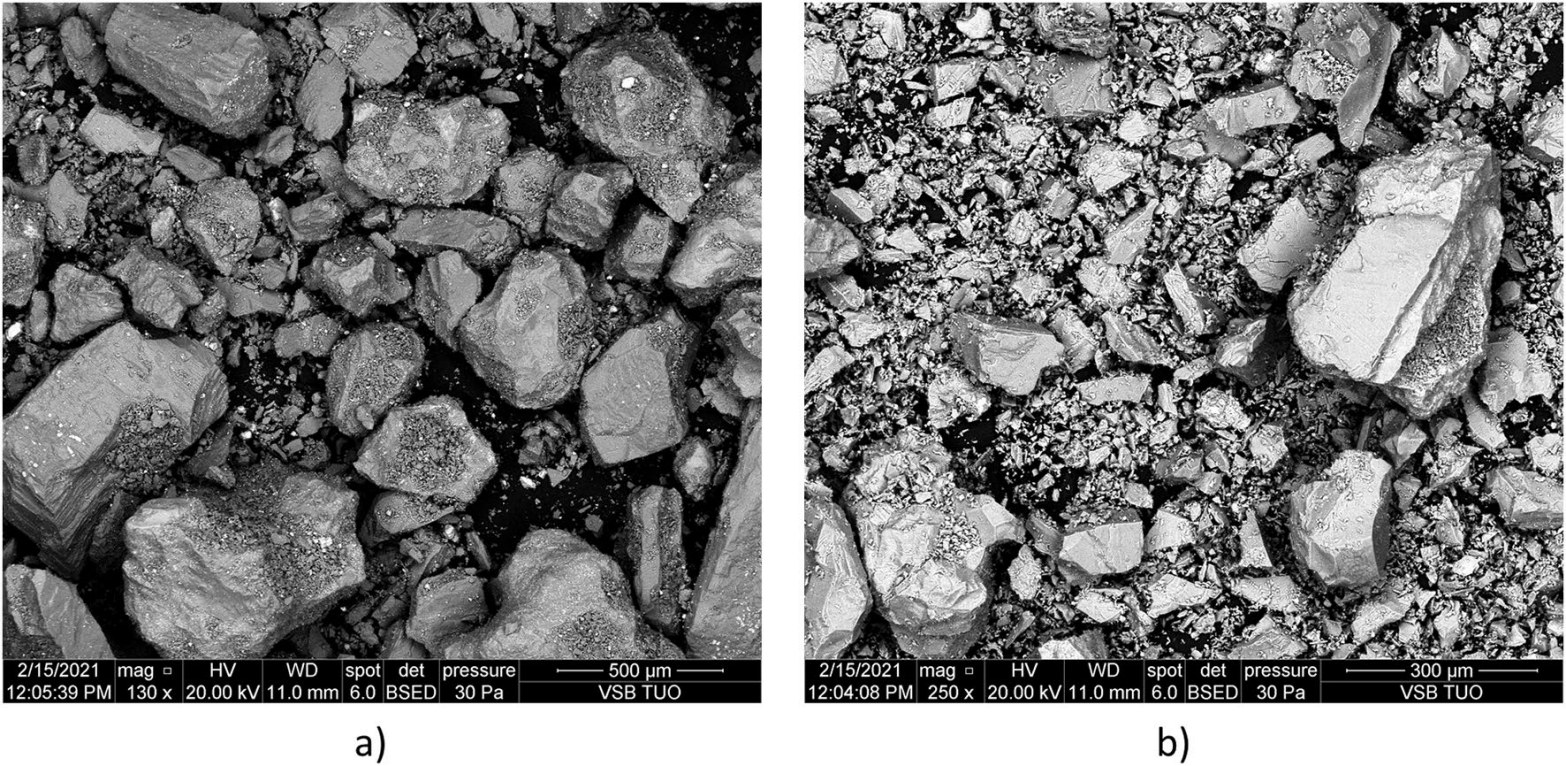
Scanning electron microscope (SEM) photos of lunar regolith simulants (**a**) LMS-1, and (**b**) LHS-1.

For both simulants, all particles were smaller than 500 µm. LHS-1 had smaller particles than LMS-1, as shown in Table 2. The parameters d_mean_, d_10_, d_50_, and d_90_ show that the difference in particle size is due to a greater representation of a larger fraction from 80 to 400 µm. The SEM photographs appropriately complement the characterization of LMS-1 and LHS-1 (Figure 6). The photographs show the faceted angular shape of the lunar regolith simulant particles.

### Shear test

The values of EAIF (*δ*), LAIF (*ϕ*), flow function *ff*_*c*_, and cohesion *c* are averaged values from 10 measurements. Table 3 shows *EAIF* (*δ*) with standard deviation (*SD*_*δ*_), its maximum value (*δ*_*max*_), and its minimum value (*δ*_*min*_), *LAIF* (*ϕ*) with standard deviation (*SD*_*ϕ*_), maximum value (*ϕ*_*max*_), and its minimum value (*ϕ*_*min*_). Table 4. shows flow function *ff*_*c*_ with standard deviation (*SD*_*ffc*_), its minimum value (*ff*_*cmin*_) and maximum value (*ff*_*cmax*_), cohesion *c* with standard deviation (*SD*_*c*_), its minimum value (*c*_*min*_) and maximum value (*c*_*max*_). As can be seen from the tables (below), the data were split into two parts, the top half with the LMS-1 specimen and the bottom half with the LHS-1 specimen. Each half of the tables is based on RST and FT4 measurements of the appropriate combination of regolith simulant and shear instrument set.

**Table 3:** Shear test results – effective angle of internal friction *δ* and linearized angle of internal friction *ϕ*.

Measuring device	Specimen and shear cell material combination	δ (°)	SD_δ_ (°)	δ_min_ (°)	*δ*_max_ (°)	*ϕ* (°)	SD_*ϕ*_ (°)	*ϕ*_min_ (°)	*ϕ*_max_ (°)
RST	LMS-1, original	43.94	1.00	42.10	45.50	42.16	1.05	40.20	43.70
	LMS-1, PLA	42.55	0.83	41.60	43.90	41.19	0.85	40.20	42.70
	LMS-1, ASA	41.52	0.65	40.60	42.40	40.06	0.65	39.20	40.90
FT4	LMS-1, original	42.81	0.38	42.33	43.75	40.36	0.34	39.77	41.08
	LMS-1, PLA	40.07	0.68	39.09	41.10	36.87	0.74	35.41	37.80
	LMS-1, ASA	40.92	1.07	39.54	42.95	37.64	0.87	36.61	39.25
RST	LHS-1, original	41.74	0.18	41.50	42.10	40.03	0.15	39.80	40.30
	LHS-1, PLA	43.15	0.34	42.50	43.70	41.51	0.33	40.90	42.10
	LHS-1, ASA	42.34	0.62	41.30	42.20	40.68	0.62	39.60	41.60
FT4	LHS-1, original	39.28	0.51	38.70	40.27	36.75	0.51	36.10	37.71
	LHS-1, PLA	38.90	0.64	38.00	40.10	36.42	0.63	35.62	37.50
	LHS-1, ASA	39.34	0.74	38.07	40.40	36.74	0.59	35.62	37.56

**Table 4:** Shear test results—flow function *ff*_*c*_ and cohesion *c*.

Measuring device	Specimen and shear cell material combination	ff_c_ (−)	SD_ffc_ (−)	ff_cmin_ (−)	ff_cmax_ (−)	c (MPa)	SDc (MPa)	c_min_ (MPa)	c_max_ (MPa)
RST	LMS-1, original	12.30	0.77	11.02	13.44	414.70	33.82	356.00	463.00
	LMS-1, PLA	15.91	0.73	14.69	16.86	308.20	17.07	289.00	337.00
	LMS-1, ASA	15.51	0.89	14.24	16.65	312.60	17.81	291.00	341.00
FT4	LMS-1, original	9.33	0.79	7.99	10.43	483.18	51.73	412.23	591.27
	LMS-1, PLA	7.54	0.54	6.64	8.28	561.37	46.96	500.66	643.17
	LMS-1, ASA	7.34	0.71	6.33	8.48	576.01	58.63	494.81	665.34
RST	LHS-1, original	13.17	0.71	11.90	14.51	363.10	20.90	330.00	400.00
	LHS-1, PLA	13.40	0.79	12.08	14.54	375.30	22.82	344.00	408.00
	LHS-1, ASA	13.63	0.54	12.57	14.30	361.90	14.48	336.00	390.00
FT4	LHS-1, original	9.44	0.46	8.40	10.16	472.00	27.79	427.75	531.84
	LHS-1, PLA	9.70	0.44	8.73	10.30	448.36	21.55	421.10	497.11
	LHS-1, ASA	9.24	0.70	8.20	10.00	473.98	38.08	435.40	534.40

Comparison between RST and FT4 measuring methods should not be taken unambiguously. The values of *EAIF* (*δ*), *LAIF* (*ϕ*), *ff*_*c*_, and *c* corresponded to the size of the shear surface, being larger for the RST (8482 mm^2^) and smaller for the FT4 (1879 mm^2^)^44^. However, a comparison of standard RST and FT4 shear test methods between LMS-1 and LHS-1 regolith simulants showed slightly better flowability of LHS-1 powder. The resulting flow properties of LHS-1 are due to the larger fraction of particles below 80 µm in the powder. The smaller particles act as a lubricant that allows the larger particles to rotate into a position with the possibility of movement. Cohesion *c* shows the macro-effect of the flow properties of the powders.

The correlation between LMS-1 and LHS-1 is interesting, as the former powder had worse comparison results for different material instrument sets. The results obtained from measurements of the LMS-1 powder on RST with the original aluminium shear cell and lid had slightly worse flowability than the measurements with the PLA and ASA printed shear cells and lids. This is evident from the lower *ff*_*c*_ values and higher *EAIF* (*δ*) and *LAIF* (*ϕ*) values measured with the original set compared to the 3D printed instrument measurements. The ASA printed shear cell and lid showed the smallest standard deviation for *EAIF* (*δ*) and *LAIF* (*ϕ*). Although the standard deviation value for *ff*_*c*_ was the smallest for the original instrument set, the difference from other material sets was negligible. The most striking observation that emerged from the data comparison was the LMS-1 powder measured on FT4. The differences between *AIF* (*δ, ϕ*) were up to 3°, and the difference in *ff*_*c*_ was up to 2°. However, the slightly worse *EAIF* (*δ*) and *ff*_*c*_ of the LMS-1 measured on FT4 are due to the smaller shear vessel cross-section.

Interestingly, for the LHS-1 powder, a good corelation of the results measured on the RST was observed between all three combinations of the shear cell and lid materials. The worst *ff*_*c*_ was measured on the RST with original instruments for LHS-1 powder, but the difference from the other material instrument sets was negligible. Even in the case of LHS-1 powder measurements on FT4, a significant positive correlation was found between all three sets of measuring instruments. The *ff*_*c*_ values measured on the FT4 showed the best correlation of all measurements when comparing between different material instrument sets.

We now turn to the experimental results of cohesion *c*, which shows a correlation with the *EAIF* (*δ*), *LAIF* (*ϕ*) and *ff*_*c*_ deviations. The LMS-1 powder shows significant differences in cohesion *c* results. The measured results of cohesion *c* are somewhat counterintuitive. It is due to the reduced values that measured on the RST with the PLA and ASA printed instruments, but increased values measured on the FT4 with the PLA and ASA printed instruments compared to the original instrument sets for both devices.

### Compressibility

**Figure 7:**
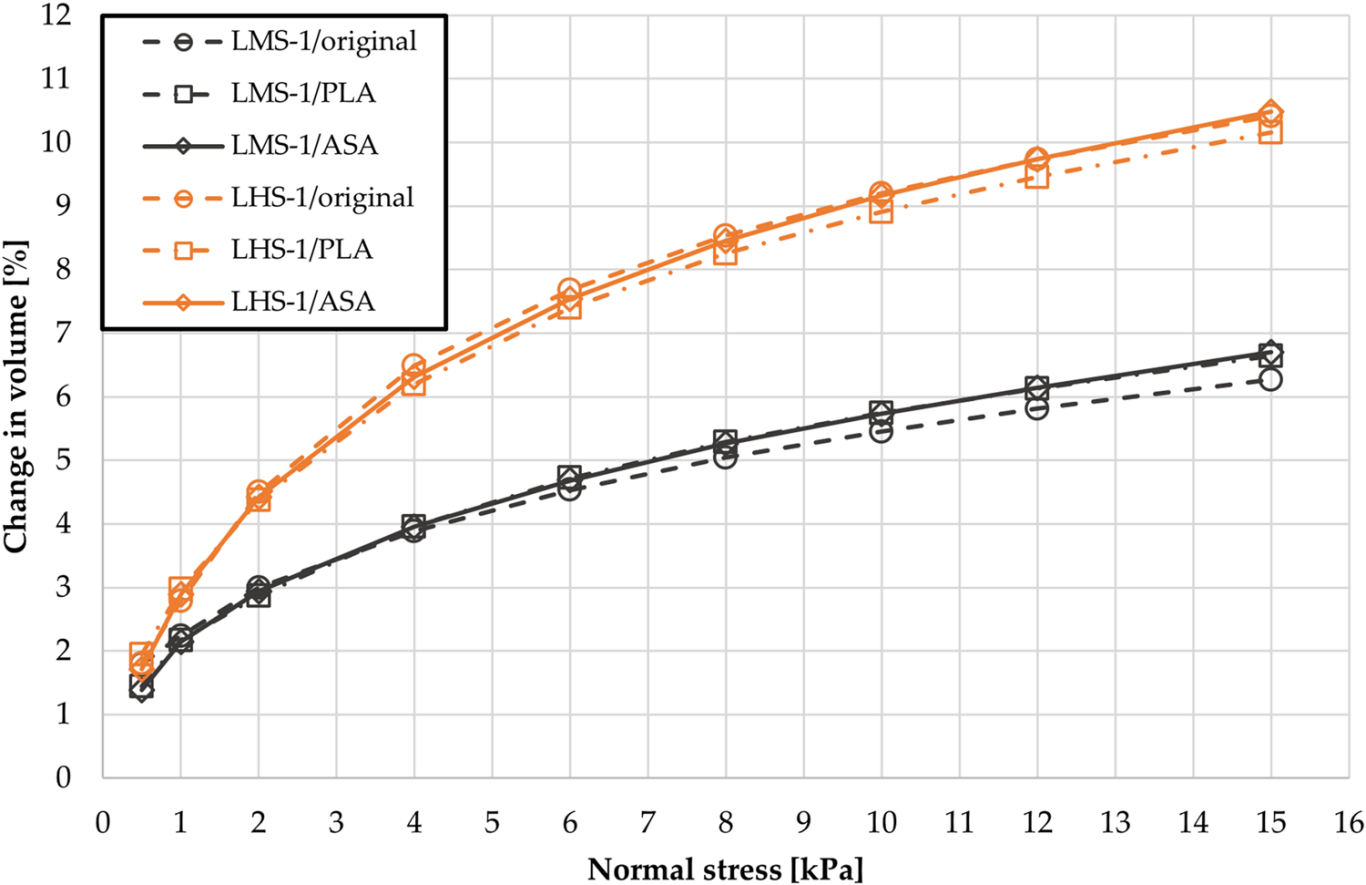
Compressibility of LMS-1 and LHS-1 powders measured by original, and 3D printed instruments.

The resulting values, shown in Fig. 7, were averaged from ten measurements of the percentage change in volume after compression. The presented compressibility curves show a high agreement when original and 3D printed instruments are compared. The LHS-1 powder with finer particles had a higher compressibility. For 15 kPa of applied normal stress, its volume changed by more than 10%. The LMS-1 powder showed compressibility of over 6% for 15 kPa of applied normal stress.

The aim of the study was to investigate the effect of 3D printed instruments and vessels on the measured compressibility values. The values showed negligible deviations as shown in Fig. 7. One unanticipated finding was that shearing of the powder after the initial conditioning had the greatest effect on the measured result. If this shearing of the powder did not take place almost perfectly in the plane, the subsequent result was noticeably different. This finding applies to both the original vessel and the 3D printed vessel. However, the 3D printed vessels had slightly poorer surface flatness in the shearing plane, which escalated the effect and the need for the perfect shear of the powder. A significant positive correlation between the diameter size of the 3D printed vented piston and the compressibility results. Due to shrinkage during 3D printing, the diameter size in the CAD model was modified to match the original vented piston after 3D printing.

### Stability index and flow rate index

**Figure 8:**
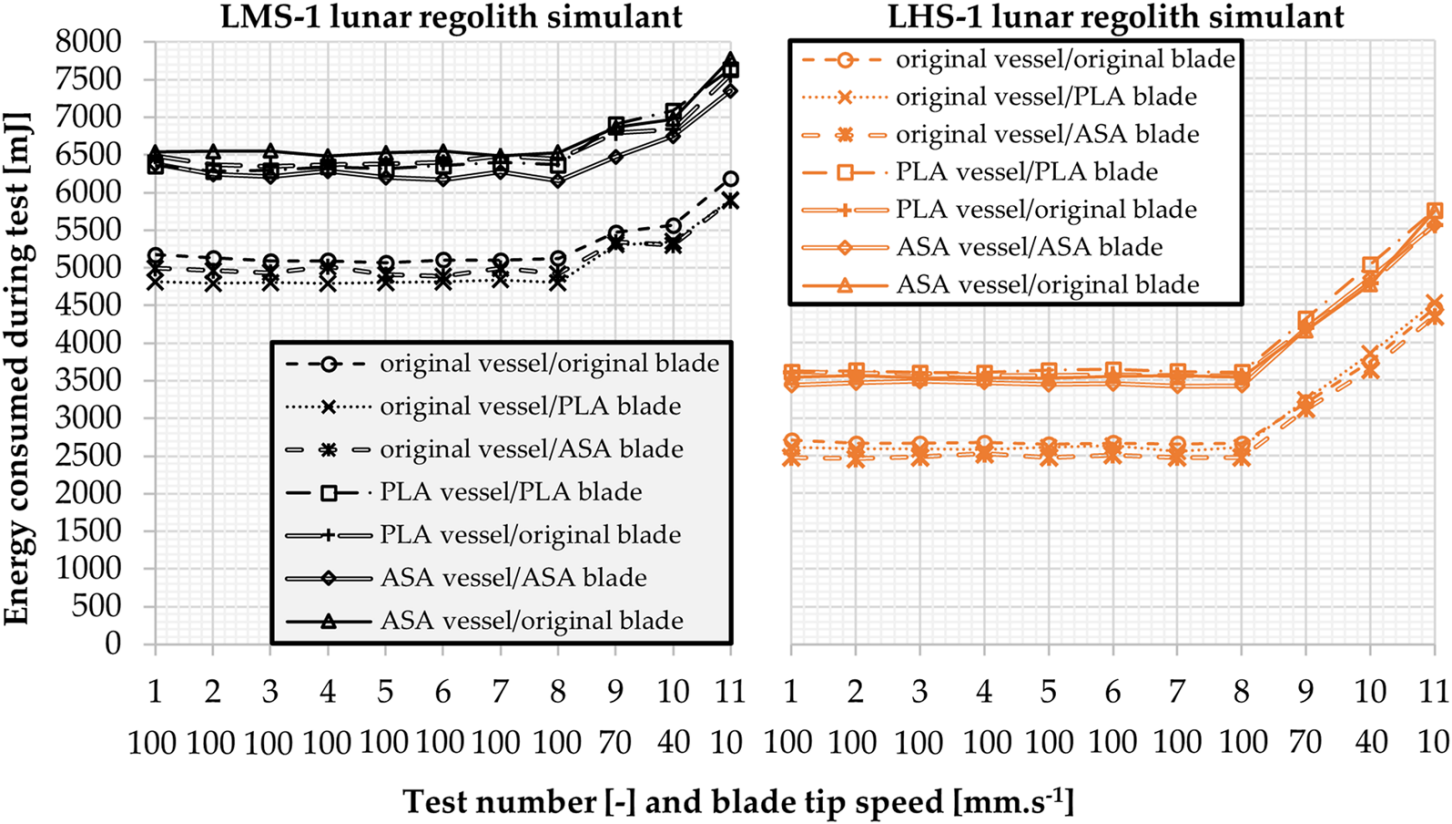
Average results for the Stability Index, Flow Rate Index and Basic Flowability Energy standardized tests—left LMS-1,—right LHS-1.

The results of the dependence of the energy consumed during the tests on the blade tip speed are shown in Fig. 8. The results are the average of 5 measurements. Both powders showed very stable values of Stability Index (*SI)*. However, the Flow Rate Index (*FRI)* values showed different behaviour for the two regoliths. The most significant difference was in the energy consumption during the tests, with the LMS-1 powder regolith showing a much higher *BFE* [mJ] than the LHS-1 powder regolith.

The largest SI deviation for the LMS-1 measurement was in the original vessel with an ASA printed blade, where *SI* = 1.00 with standard deviation less than *σ*_*SI*_ = 0.024, the minimum value of *SI*_*min*_ = 0.96 and the maximum value of *SI*_*max*_ = 1.02. The other LMS-1 measurements had the average *SI* value of 0.97 < *SI* < 1.02, a standard deviation less than *σ*_*SI*_ = 0.02, the minimum value *SI*_*min*_ = 0.95 and the maximum value *SI*_*max*_ = 1.04. Similar *SI* values were measured for the LHS-1 powder regolith. The largest deviation in SI measurement was for the ASA printed vessel with the original blade, where the average value *SI* = 1.01 had a standard deviation less than *σ*_*SI*_ = 0.038, the minimum value *SI*_*min*_ = 0.97 and the maximum value *SI*_*max*_ = 1.08. For the other LHS-1 measurements, the average SI was less than 0.97 < *SI* < 1.02, standard deviation smaller than *σ*_*SI*_ = 0.029, the minimum value *SI*_*min*_ = 0.94 and the maximum value *SI*_*max*_ = 1.03.

Although *SI* showed similar stability for both powders, the *FRI* showed different behaviour. The largest deviation in the *FRI* measurement of the LMS-1 powder regolith was for the original vessel with ASA printed blade, where the average value of *FRI* = 1.20 with the standard deviation less than *σ*_*FRI*_ = 0.034, the minimum value *FRI*_*min*_ = 1.16 and the maximum value *FRI*_*max*_ = 1.24. The other LMS-1 measurements had the average *FRI* value of 1.17 < *FRI* < 1.23, the standard deviation less than *σ*_*FRI*_ = 0.015, the minimum value *FRI*_*min*_ = 1.15 and the maximum value *FRI*_*max*_ = 1.23. The *FRI* measurement of the LHS-1 powder regolith showed a difference, with the average *FRI* value ranging 1.56 < *FRI* < 1.76, the standard deviation being less than *σ*_*FRI*_ = 0.036, the minimum value being *FRI*_*min*_ = 1.54 and the maximum value being *FRI*_*max*_ = 1.81. The LMS-1 powder showed less sensitivity than the LHS-1 powder.

The Basic Flowability Energy (*BFE*) values showed the dependence of the blade tip speed of 100 mm.s^-1^ on the energy consumption during the test 7. A strong correlation was found between the vessel fabrication technique and the energy consumption during the test. Due to the layering of the vessel that is formed during the 3D printing, the energy consumption increased. A more detailed description of modification and 3D printing was mentioned in section Fused Filament Fabrication printed equipment.

The BFE measurements of the LMS-1 powder with the original vessel showed average values in the range of 4 843 mJ < *BFE* < 5 092 mJ, with the standard deviation *σ*_*BFE*_ = 200 mJ, the minimum value *BFE*_*min*_ = 4 692 mJ and the maximum value *BFE*_*max*_ = 5 291 mJ. When measured in the 3D printed vessels, the *BFE* values increased by 1 435 mJ to average values in the range of 6 277 mJ < *BFE* < 6 487 mJ, with the standard deviation *σ*_*BFE*_ = 172 mJ, the minimum value *BFE*_*min*_ = 6 133 mJ and the maximum value *BFE*_*max*_ = 6 777 mJ.

The measurements of LHS-1 powder showed a similar increase in the energy consumption for the non-original vessel. The average *BFE* values for the original vessel ranged 2 473 mJ < *BFE* < 2 658 mJ, with the standard deviation *σ*_*BFE*_ = 81 mJ, the minimum value *BFE*_*min*_ = 2 382 mJ and the maximum value *BFE*_*max*_ = 2 749 mJ. The average *BFE* values increased by 976 mJ when measured in the 3D printed vessels, giving a range of 3 422 mJ < *BFE* < 3 617 mJ, with the standard deviation *σ*_*BFE*_ = 162 mJ, the minimum value *BFE*_*min*_ = 3 291 mJ and the maximum value *BFE*_*max*_ = 3 837 mJ. The average *BFE* values measured in the 3D printed vessels could be improved by treating the inner surface of the vessel after 3D printing. However, this comparison showed importance of the vessel material and fabrication method.

## Conclusions

The aim of this study was to investigate the possibility of using measuring instruments made by material extrusion 3D printing method to measure selected mechanical-physical properties of bulk materials in extreme conditions. This study showed that the measurement methods used, such as Schulze’s ring shear test, FT4’s shear test, FT4’s compressibility test, and FT4’s Flow Rate and Stability tests, can be carried out using interchangeable measuring instruments. However, this problematic has its issues, and it is important to be clear about the pros and cons and to know which results are affected and how. Due to the different mechanical properties of the materials used for the measuring instruments, between the original (steel or glass) and the 3D printing (plastics), the question of the influence on the measurement process arises. Specifically, this will relate to surface roughness and stiffness. Surface roughness and stiffness are adjustable within certain limits by the 3D printing process. Further, tribocharging can be a crucial parameter for some types of materials, affecting the measurement results, for example, for very light and small grain materials. In the case of our research, no significant effect of tribocharging was assumed. The Schulze’s shear cell and lid^32^ was at least 2.5 times lighter than the original measuring instruments when using PLA printed instruments, and more than 3 times lighter than the originals when using the ASA printed instruments. The 3D printed Schulze’s shear cell and lid could be even lighter, due to the dependence on the percentage of the infill that could go down with less normal stress. The 3D printed FT4’s vessels were more than 2.6 times lighter than the original vessel assemblies^42^. The 3D printed FT4’s measuring instruments were more than 4 times lighter than the original measuring instruments, and the ASA printed blade and shear head were even more than 10 times lighter.

As for the choice of the 3D printing method, we used FFF method because we opted for a cheap and simple option. The FDM method was patented by company Stratatys. In comparison to FFF, FDM printing is more precise, has better surface quality, and the parts have better mechanical properties. The disadvantage is the high price and necessity to always use a soluble support material. Most of the advantages are due to the enclosed printing chamber which allows the internal temperature to be maintained (for example: 90 °C for ABS). This type of printing environment is called hot-hot-hot (nozzle-air-heated bed) as the opposite to the FFF hot–cold-hot environment. It leads to better mechanical properties, where the adhesion between layers is strengthened and warping and curling of the printed parts are prevented^16^.

The standardized methods of the particulate material measurement are very difficult to replicate accurately in hard-to-reach locations, such as non-laboratory environments in the field, or on extra-terrestrial bodies. These types of measurements will always be affected and deviated from the laboratory measurements. However, the methodology has fundamental foundations that should be preserved. These fundamentals are based on software and machine hardware, such as steel movement guidelines, strain gauges, material weighing, and programme evaluation, and should not be changed or modified to maintain the ability to compare the results with these laboratory measurements.

As mentioned above, the lunar simulant powders are terrestrial based and simulate mineralogy, chemical composition, and particle size distribution. However, due to bad simulation of particle shape and weathering, the results cannot be used as information to design storage, handling, and process equipment elsewhere then on the planet Earth.

The first non-laboratory and extra-terrestrial in situ measurements would be indicative. However, further measurements will provide the opportunity to compare measured results, allowing for the design and optimisation of process and handling equipment on extra-terrestrial bodies. The research leads to the following major conclusions:

- The blade that was 3D printed with FFF technology is not a perfect copy. The manufacturing technology did not have a high enough precision to produce a geometrically identical blade curvature. The surface smoothness was not good and surface layering of the curved part could be seen by a naked eye. As mentioned above, the blade angle was modified from the original. Modification was made to maintain mechanical load during measurement and to measure similar torsional resistance. The smoothing of the blade surface slightly changed measured values of stability index (*SI*) and flow rate index (*FRI*). However, after two initial measurements the measured values stabilized, and those values of initial tests were not included into results.
- The PLA filament was chosen as a cheap and an easy-to-print filament for the evaluation of 3D printed measuring instruments, such as design, functionality, printability. Although the ASA filament is harder to print and more expensive than PLA, with its mechanical properties, UV and colour resistance, temperature resistance and lower density than PLA made it a great choice for the laboratory setup in a harsher environment. However, in extreme environments, the process equipment should be designed for the actual mechanical-physical properties of the bulk material. Therefore, better filament material might be needed. Currently, filaments made of PolyEtherEtherKeton (PEEK), which has strength-to-weight ratio comparable to stainless steel and exceptional thermal properties. The material can withstand thermal-cycling in vacuum chambers used for space-qualification tests, radiation, or wear. The material requires fused deposition modelling (FDM) printing technology with nozzle temperatures around 400 °C, and it is used in space industry, aviation, oil industry, and advanced mechanical engineering applications.
- As mentioned above, the standard compressibility test is highly dependent on the proper shear of the particulate material in the vessel after the initial conditioning. The flatness of the surface is dependent on the printing method and can lead to different compressibility values if the shear is not performed correctly in the plane. Furthermore, the diameter of the 3D printed piston was modified in CAD model due to the shrinkage to match the original diameter after 3D printing.
- The Flow Rate Index (FRI) and Stability Index (SI) measurements show dependence on vessel material and on the vessel manufacturing method. However, only the Basic Flow Energy (BFE) was negatively affected by the increase in energy consumed during the tests. The FRI and the SI results remained stable and repeatable when 3D printed vessels were used and energy consumption increased during tests.
- For the 3D printed measuring instrument, a suitable filament material should be used to have sufficient chemical resistance for bulk material that is measured. Furthermore, a suitable filament material should be used to withstand the mechanical load during measurement. Finally, the filament material should have UV and radiation stability for the environment in which the particulate material is measured.

To summarize, the biggest disadvantages of the 3D printed measuring instruments were higher measurement error rate due to the mechanical errors (FT4’s blade, shear head and vented piston interference), slightly higher measurement deviations, and manufacturing imperfections that can be reduced by additional post-processing after printing.

The biggest advantages of the 3D printed measuring instruments were the lower weight, the ability to pre-print a set or to print on the spot, to replace a damaged part with a new 3D printed part on-demand if extremely fast results are needed or due to the logistical unavailability, customization of the standardized tests for better understanding the behaviour of the particulate materials, and cheaper manufacturing cost (at least 30 times lower in the case of the FFF printing method).

Overall, this study reinforced the idea that the measurement of mechanical-physical properties in locations where light weight is of essence, where interchangeable instruments are needed to measure approximate values, and where a quick result is more important than an extremely precise result. The 3D printing method is significantly more versatile for on-site tool construction than conventional methods of machining steel etc. This allows us to react quickly to specific extreme conditions, which cannot always be accurately predicted.

## Data Availability

The datasets used and/or analyzed during the current study available from the corresponding author on reasonable request.
